# Pregabalin and Duloxetine in Patients with Non-Nociceptive Pain: A Narrative Review Exploring the Pharmacological Effects of This Combination

**DOI:** 10.3390/ph18101434

**Published:** 2025-09-25

**Authors:** Gianmarco Marcianò, Maurizio Evangelista, Cristina Vocca, Vincenzo Rania, Caterina Palleria, Maria Cristina Caroleo, Riccardo Torta, Luca Gallelli

**Affiliations:** 1 Department of Health Science, University Magna Graecia of Catanzaro, 88100 Catanzaro, Italy; gianmarco.marciano3@gmail.com (G.M.); cristina_vocca@live.it (C.V.); mariacristina.caroleo@unicz.it (M.C.C.); 2 Department of Anesthesia, Resuscitation and Pain Therapy, Sacred Heart Catholic University, 00100 Rome, Italy; maurizio.evangelista@unicatt.it; 3 Operative Unit of Clinical Pharmacology and Pharmacovigilance, Renato Dulbecco University Hospital, 88100 Catanzaro, Italy; raniavincenzo1@gmail.com (V.R.); palleria@unicz.it (C.P.); 4 Research Center FAS@UMG, Department of Health Science, University Magna Graecia of Catanzaro, 88100 Catanzaro, Italy; 5 Department of Neurosciences “Rita Levi Montalcini”, University of Turin, Via Cherasco 15, 10126 Turin, Italy; riccardo.torta@unito.it; 6 Medifarmagen, Renato Dulbecco University Hospital, 88100 Catanzaro, Italy

**Keywords:** pregabalin, duloxetine, neuropathic pain, nociplastic pain, safety

## Abstract

Both neuropathic and nociplastic pain (non-nociceptive pain) are characterized by a similar pattern of clinical symptoms, including numbness, dysesthesia, tingling, and pricking. Whereas nociplastic pain results from altered nociception without indication of tissue damage or a somatosensory system lesion, neuropathic pain is caused by a disease or lesion affecting the somatosensory system. The available therapeutic options consist of antiepileptic drugs, antidepressants, and muscle relaxants. Unfortunately, symptoms are often refractory, and increasing drug dosage may lead to adverse events. In this narrative review, we searched PubMed, MEDLINE, Cochrane, and EMBASE databases from their inception up to 26 July 2025, using the key words “duloxetine,” “pregabalin,” and then ‘‘combination,’’ “nociplastic pain,” “neuropathic pain,” “efficacy,” “safety,” “pharmacology,” “pharmacokinetic,” and “pharmacodynamic.” We evaluated the role of combination therapy with duloxetine, a serotonin–norepinephrine reuptake inhibitor, and pregabalin, an antiseizure medication that acts on voltage-gated calcium channels α2δ subunit, in patients with neuropathic or nociplastic pain. The literature data indicate that combination therapy has synergistic effects, leading to fewer adverse events in specific categories of patients. Available evidence showed that combination therapy is generally not inferior to monotherapy, with slight differences in safety outcomes depending on supplementation, drug labels, and titration. These results indicate that even if not superior, combination therapy may be an alternative to monotherapy in selected patients: those who experience side effects from higher dosages of duloxetine or pregabalin and for whom symptom relief from dose reduction alone is not possible; those who use medications that interact with duloxetine; those who suffer from anxiety–depression, where pain is closely linked to mental symptoms; and those who have central neuropathic pain (often refractory).

## 1. Introduction

Neuropathic pain is defined by the International Association for the Study of Pain (IASP) as “pain that arises as a direct consequence of a lesion or diseases affecting the somatosensory system” [1] that could be related to diabetic neuropathy, low back or cervical pain with myelopathy, herpetic, and trigeminal neuralgia [2,3,4].

The therapeutic options to manage neuropathic pain include antidepressants (e.g., amitriptyline, duloxetine), antiepileptics (pregabalin, gabapentin, carbamazepine), capsaicin or lidocaine patches, and acetyl-L-carnitine. Antidepressants and gabapentinoids have the stronger evidence level as first-line. Capsaicin and lidocaine have a weak recommendation as second-level [2,5]. Furthermore, other therapeutic options like botulin toxin (BTX-A), opioids, and repetitive transcranial magnetic stimulation are considered for third-line (weak recommendation) [5].

Conversely, nociplastic pain is defined as pain “arising from altered nociception despite no clear evidence of actual or threatened tissue damage causing the activation of peripheral nociceptors or evidence for disease or lesion of the somatosensory system causing the pain” [1].

Complex regional pain syndrome (CRPS), bladder pain syndrome, fibromyalgia, and chronic pelvic pain are among the clinical conditions characterized by central sensitization that results in nociplastic pain [6,7].

The onset of nociplastic pain might be related to (i) central sensitization (altered balance between excitatory and inhibitory neurotransmitters and changes in synaptic transmission); (ii) supraspinal (altered brain activity and emotional and cognitive factors), spinal (decreased descending inhibition), and/or peripheral mechanisms (change in sodium channel activity and overactivation of the sympathetic nervous system); and (iii) the immune system (neuroinflammation) [8,9].

Drugs commonly used in patients with nociplastic pain are antidepressants and antiepileptics that are also able to manage anxiety/depression and insomnia often related to this clinical condition. Serotonin–norepinephrine reuptake inhibitors (SNRIs), especially duloxetine, are effective antidepressants in treating nociplastic pain (fibromyalgia) and neuropathic pain (sciatica, back pain, diabetic neuropathy) [10,11]. Nevertheless, the low number of trials for nociplastic pain, alongside their small sample sizes and associated bias renders new studies necessary. Despite some trials showing a certain degree of efficacy for venlafaxine, the higher dosage needed to achieve noradrenergic effect and the inferiority in comparative studies renders duloxetine the best option. Tricyclic antidepressants have shown efficacy in managing neuropathic pain, but they do not always display superiority vs. a placebo. Furthermore, the possible onset of side effects related to adrenergic, muscarinic, and histaminergic receptors is an important concern [12]. Few antiepileptic drugs have shown concrete efficacy in managing neuropathic and nociplastic pain. Gabapentinoids and carbamazepine are the compounds with more clinical evidence [13]. The use of carbamazepine is typically restricted to treating trigeminal neuralgia due to its high risk of drug interactions and side effects, which include hepatotoxicity and cutaneous reactions [14]. Gabapentinoids have shown efficacy in both nociplastic and neuropathic pain, with pregabalin being tested in most of the studies. This drug class shows better safety when compared to other medications [2]. However, only 35% of people achieved a 50% pain relief in clinical studies, compared with 21% for a placebo. Topical clinical agents like capsaicin and lidocaine patches are used for specific indications like post-herpetic neuralgia and diabetic neuropathy [2]. These compounds display a minor drug-interaction rate but do not exert a systemic effect. Other therapies like cannabinoids are less common and still face several ethical problems [12].

However, other drugs, such as bisphosphonates, particularly neridronate, can be used to reduce bone marrow edema in CRPS patients [15].

Pregabalin and duloxetine play a key role in the management of neuropathic and nociplastic pain (non-nociceptive pain). They are administered both as a monotherapy and in combination, obtaining synergistic effects [16,17,18]. In this narrative review, we summarized the data on clinical efficacy and safety of pregabalin and duloxetine as a combination therapy.

## 2. Methods

In agreement with our recent papers [2,4,19,20], we included reviews, randomized clinical trial (RCTs), or meta-analyses that evaluated the effect of duloxetine, pregabalin and their combination in nociplastic and neuropathic pain. We searched PubMed, MEDLINE, Cochrane, and EMBASE databases from their inception up to 26 July 2025, using the key words “duloxetine,” “pregabalin,” and then ‘‘combination,’’ “nociplastic pain,” “neuropathic pain,” “efficacy,” “safety,” “pharmacology,” “pharmacokinetic,” and “pharmacodynamic,” combined with Boole’s logical operators.

The records were first screened by title/abstract, and then full-text articles were retrieved for eligibility evaluation. The reference lists of previous reviews and the included studies were also examined. The patients enrolled in clinical studies, cited in the manuscript, were all affected by nociplastic and neuropathic pain. In this review, we included peer-reviewed original research articles and guidelines analyzing combination therapy in patients ≥ 18 years with a diagnosis of neuropathic or nociplastic pain. On the other hand, we excluded manuscripts that enrolled patients younger than 18 years old or those that were not written in English.

## 3. Duloxetine

### 3.1. Duloxetine Pharmacodynamics

**Figure 1:**
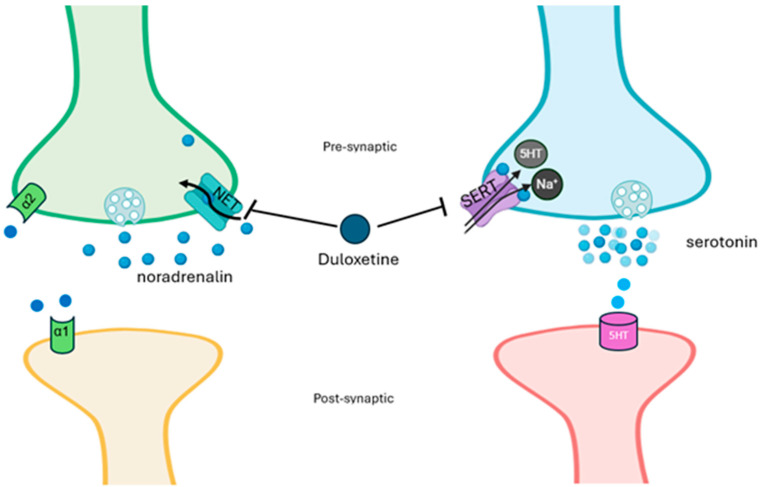
**Mechanism of action of duloxetine.** Duloxetine acts by inhibiting the sodium-dependent serotonin transporter (SERT) and the norepinephrine transporter (NET), resulting in increased levels of both norepinephrine and serotonin in the synaptic cleft and increased receptor activation. Presynaptic alpha-2 receptors stimulated from the high levels of synaptic norepinephrine reduce the secretion of norepinephrine with a feedback mechanism.

Duloxetine is a serotonin–norepinephrine reuptake inhibitor (SNRI) [21]; it enhances neurotransmitter activity by central neurons in the brain and spinal cord (Figure 1), thereby enhancing the function of the descending inhibitory pathway and restoring the balance of the descending inhibitory and facilitation systems [22].

Through this mechanism, duloxetine inhibits pain perception and modulates emotional components. Therefore, duloxetine represents the first choice for patients with anxiety/depression suffering from pain [18,23]. Duloxetine [24,25,26] inhibits neuroinflammation occurring in both pain and emotional states through a decrease in interleukin (IL)-8, IL-12, and interferon (IFN)-γ [27]. Costa and Guidotti documented that antidepressants promote the synthesis of allopregnanolone [28], an anxiolytic and antidysphoric neurosteroid involved in both emotional and pain aspects; this effect is related to the activation of gamma-aminobutyric acid (GABA) A receptor and inhibition of glutamate pathways [29,30] and does not induce the development of side effects. In humans, blood levels of allopregnanolone are estimated to be inversely associated with both low back pain and chest pain [31].

### 3.2. Duloxetine Pharmacokinetics

Considering its pharmacokinetic properties (Table 1), duloxetine should be used with caution in poly-treated patients with blood hypertension and with eGFR < 30 mL/min.

## 4. Pregabalin

### 4.1. Pharmacodynamics

**Figure 2:**
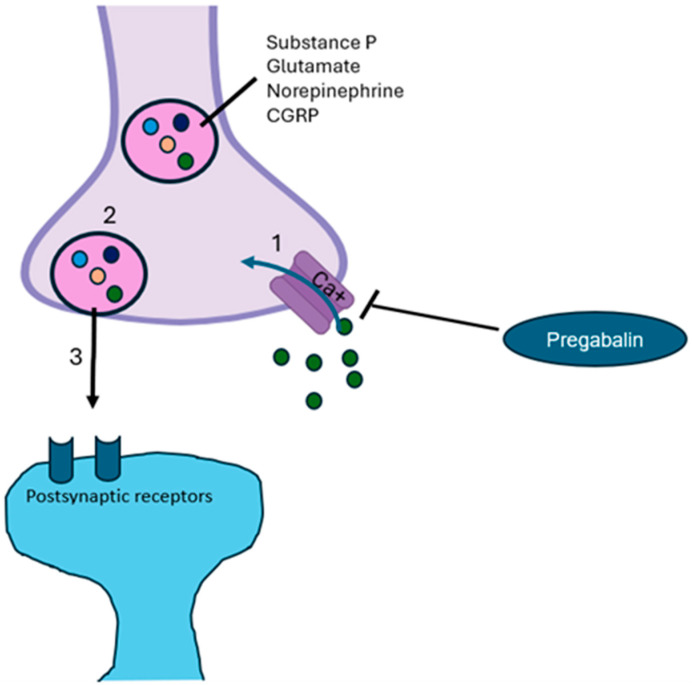
**Mechanism of action of pregabalin.** Pregabalin inhibits calcium channels activity, avoiding depolarization and consequential release of excitatory neurotransmitters [32,40]. CGRP, calcitonin gene-related peptide.

Pregabalin, which enhances GABA neurotransmission and reduces glutamate levels, modulates not only pain but also emotional components (i.e., anxiety and insomnia) [35]. A mechanism that potentially contributes to mood alterations is reduced hippocampal neurogenesis (ahNG). Chronic pain may also induce profound changes in hippocampal plasticity; therefore, pregabalin, acting on the α2δ subunit, can act to restore neuronal plasticity [36]. All of these mechanisms support the fact that pregabalin’s effect on cortical excitability occurs particularly under basal neuronal hyperexcitability (such as chronic pain) through an intracortical inhibition in specific neural networks linked to emotional states and pain [36,37]. Pregabalin also promotes the production of IL-10 and beta-endorphins and inhibits both the reactive microglia and the co-expression of proinflammatory cytokines (such as IL-2 and IL-6) [38]. Ellergezen et al. [37], in 35 women with fibromyalgia syndrome treated for 3 months with pregabalin (150 mg/day), reported a significant decrease in plasma levels of proinflammatory cytokines (IL-2, IL-6, IL-12, IL-17, interferon-gamma, and tumor necrosis factor-alpha) compared to non-treated women with fibromyalgia (*n* = 30) and healthy women (*n* = 25), suggesting that pregabalin modulates the immune system in neuroinflammatory diseases, such as nociplastic pain and anxiety. Pregabalin, is an oral antiepileptic drug targeting the α2δ calcium voltage-gated channels subunit [39] (Figure 2).

**Figure 3:**
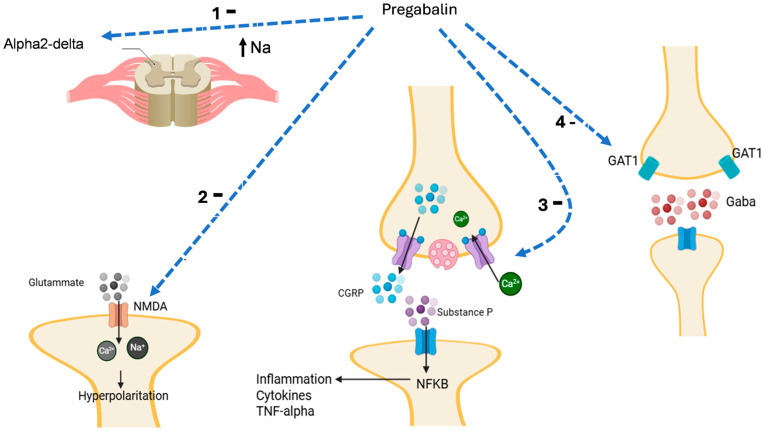
**Pregabalin inhibits** (1) alpha-2delta channel, reducing cell activation and hyperpolarization with the release of mediators; (2) N-methyl-D-Aspartate (NMDA) receptor, reducing glutamate activity, cell hyperpolarization, and the release of neurotransmitters; (3) voltage-gated calcium channel (VGCC) activity, reducing the release of calcitonin gene-related peptide (CGRP) and substance P release with inhibition of the activation of nuclear factor-kappa B (NF-κB); (4) gamma-aminobutyric acid (GABA) transporter type-1 (GAT1) increasing the GABA synaptic levels with inhibition of cell activity. TNF, tumor necrosis factor. Moreover, gabapentin also inhibits the presynaptic release of GABA, inducing an increase in the glutamate level, which in turn causes the release of norepinephrine (NA) in the spinal cord, which stimulates descending inhibition. Light blue dashed arrow: inhibition; black arrow: activation or ion movement.

Owing to these mechanisms, pregabalin reduces the symptoms of neuropathic pain (e.g., numbness, prickling, tingling, and burning), thus representing an effective therapeutic option in patients with low back pain, diabetic neuropathy, generalized anxiety, and insomnia [41]. Adjunctive pregabalin mechanisms are summarized in Figure 3.

### 4.2. Pharmacokinetics

Considering its favorable pharmacokinetic profile (Table 1), pregabalin represents a safe treatment in poly-treated patients with non-nociceptive pain [42,43].

To minimize the development of adverse drug reactions (i.e., dizziness, drowsiness, confusion, and erectile dysfunction) [40,44,45], pregabalin should be started at a low dosage (50–75 mg/daily at the night) and then gradually increased every 5 days upon evaluating both its safety and efficacy. A more gradual titration or a lower dose is feasible, particularly in patients susceptible to side effects and with estimated glomerular filtration rate (eGFR) reduction [40,46].

A comparison of the two drugs is available in Table 2.

## 5. Preclinical Evidence

Contradictory results emerged from preclinical studies.

Tripathi et al. [16], in their experimental model of diabetic neuropathy induced by streptozotocin, highlighted the complete efficacy on neuropathic pain of duloxetine plus pregabalin, duloxetine plus amitriptyline, and amitriptyline plus pregabalin. Duloxetine plus pregabalin was the combination achieving faster clinical effectiveness. However, its efficacy had almost the same efficacy of duloxetine alone.

Rodrigues et al. [49] tested the combination of pregabalin and duloxetine (vs. amitriptyline + pregabalin and pregabalin alone) in an experimental model of neuropathic pain induced by sciatic nerve constriction. The authors found no significant pharmacokinetic influences of the two compounds. No significant effect of the combination in comparison to pregabalin monotherapy was found. Nevertheless, the authors observe that their results are partially in conflict with other studies (both human and preclinical) that show the effectiveness of the combination.

The work by Nozawa and colleagues [50] on an experimental model of L5 spinal nerve ligation added interesting information to the aforementioned results. The authors tested pregabalin, duloxetine, venlafaxine, tramadol, celecoxib, and their combination, measuring pain threshold after administration. Duloxetine plus pregabalin displayed an additive effect but not a synergistic effect, differently from pregabalin plus tramadol.

In consideration of these results, scientists are trying to combine alternatively the two drugs with other compounds including the muscle relaxant tolperisone. Tolperisone + pregabalin showed a good efficacy and safety profile in a model of neuropathic pain induced by partial sciatic nerve ligation [51].

## 6. Clinical Applications

Pregabalin and duloxetine represent the first line of treatment in patients with neuropathic pain [2]. Since several studies reported their efficacy with respect to control groups as single components (Table 3), very few studies have documented their efficacy as a combination treatment.

Combination therapy can improve clinical effects (synergic effects) reducing the development of adverse drug reactions (dosage decrease) [58,59,60].

### 6.1. Efficacy

In a double-blind, randomized, parallel group study, Tesfaye et al. [61] compared the effects of duloxetine 60 mg plus pregabalin 300 mg in patients with diabetic neuropathy in patients previously treated for 8 weeks with pregabalin 600 mg or duloxetine 60 mg (804 patients for initial therapy and 339 for combination therapy). The primary outcome (Brief Pain Inventory Modified Short Form [BPI-MSF] 24 h average pain change after combination/high-dose therapy) was analyzed, comparing combination with high-dose monotherapy, whereas secondary endpoints were BPI-MSF severity items, response rates, and comparison of the two drugs for average pain. Combination therapy and monotherapy displayed a similar efficacy for pain control (BPI-MSF mean change: combination: −2.35; high-dose monotherapy: −2.16; *p* = 0.370). Similar results were obtained also for secondary endpoints, which, however, were in favor of combination therapy. This trial showed non-inferiority when compared to the use of monotherapy.

In a multicenter, double-blind, randomized crossover trial, OPTION-DM [62], diabetic neuropathic patients (140 subjects) with a numerical rating scale (NRS) ≥ 4 were randomized to receive amitriptyline, duloxetine, or pregabalin for 6 weeks; patients that experienced a scarce pain relief (mean NRS 6.6) were supplemented for 16 weeks with amitriptyline (pregabalin–amitriptyline) or pregabalin (amitriptyline–pregabalin and duloxetine–pregabalin). Concerning monotherapies, amitriptyline was titrated to a maximum of 75 mg per day, duloxetine to 120 mg per day, and pregabalin to 600 mg per day or less, depending on eGFR. The authors clarify in an appendix that duloxetine–pregabalin was administered with a titration phase of 2 weeks (week 1 duloxetine 30 mg + pregabalin 75 mg; week 2 duloxetine 30 mg + pregabalin 150 mg) and a maintenance phase of 4 weeks (duloxetine 30 mg twice daily + pregabalin 150 mg twice daily). Nevertheless, dose variations were made according to the maximum tolerated dose and pain assessment. After treatment, patients showed pain relief (NRS 3.3) in all three arms. Combination treatment resulted in being non-inferior to monotherapy.

In a sub-analysis of the OPTION-DM study [63], the authors reported no significant differences in terms of costs and efficacy for each group of treatment, suggesting that the choice of treatment may be related to the patient’s preference.

Krishnaprasad et al. [17] used a fixed dose of pregabalin plus duloxetine (50 + 20 mg, gradually titrated to 75 + 30 mg) for 7 weeks in patients with moderate–severe neuropathic pain and compared it to pregabalin 75–300 mg/daily, without a statistically significant difference between the two treatments in terms of efficacy and safety. This trial confirmed the results of the previously mentioned assessment of non-inferiority.

Saxena et al. [64], in a randomized, double-blind study trial on 34 patients with diabetic neuropathy, compared the effects of pregabalin–duloxetine (75 mg + 30 mg once daily for patients < 80 kg and bid for patients > 80 kg) vs. pregabalin alone (75 mg twice daily) administered for 4 weeks. They also analyzed expression of the peroxisome proliferator-activated receptor (PPAR) γ and protein kinase B (Akt), two proteins involved in neuropathic pain in patients with diabetic neuropathy. The authors showed that the treatment with pregabalin–duloxetine significantly decreased pain symptoms vs. pregabalin alone at 2 (*p* = 0.008) and 4 weeks (*p* = 0.002). Furthermore, the combination of pregabalin–duloxetine significantly increased the expression of PPAR γ (*p* < 0.001) but not Akt vs. monotherapy (each one significantly different from baseline); since Akt was significantly related to pain decrease in the combination group (*p* < 0.05).

Wang et al. [65], in a double-blind, randomized, crossover trial in 220 patients with post-herpetic neuralgia, evaluated the effects of duloxetine plus pregabalin (*n* = 110) and amitriptyline plus pregabalin (*n* = 110) on pain relief (evaluated through NRS), sleep (evaluated through Pittsburgh Sleep Quality Index), depression (evaluated through 17-item Hamilton Depression Rating Scale), and quality of life (evaluated through 36-Item Short Form Health Survey). The starting doses were, respectively, duloxetine 30 mg once a day before bedtime; amitriptyline 25 mg once a day before bedtime; pregabalin 150 mg twice daily. The doses of duloxetine or amitriptyline were eventually increased every 2 weeks based on pain assessment (maximum daily doses: duloxetine 90 mg, amitriptyline 75 mg). For participants who experienced adverse reactions, the dosage was adjusted. Acetaminophen was used as a rescue medication. During the 16-week study period, both treatments reduced NRS levels (*p*  <  0.001) and improved sleep, mood, and quality of life, without significant differences between the two groups. Duloxetine plus pregabalin can improve 52%, 24%, and 7% of good, moderate, and mild pain, respectively; amitriptyline plus pregabalin can reduce 48%, 21%, and 9% of good, moderate, and mild pain, respectively.

Gilron et al. [66], in 41 patients with fibromyalgia, compared the effect of pregabalin–duloxetine vs. monotherapy (placebo, pregabalin, duloxetine) for 6 weeks. Their study used a flexible dosing scheme, only considering a ceiling for pregabalin (450 mg daily) and duloxetine (120 mg daily). The average doses in combination therapy were generally inferior to those of monotherapy. The authors evaluated daily pain as the primary outcome, and the Fibromyalgia Impact Questionnaire, SF-36 survey, global pain relief, the Medical Outcomes Study Sleep Scale, the Beck Depression Inventory (BDI-II), and adverse events as secondary outcomes. The combination showed its significant superiority only towards pregabalin and the placebo in the primary outcome. Concerning secondary outcomes, the combination gained statistical significance for global pain relief, SF-36 scores, and the Fibromyalgia Impact Questionnaire (vs. all the comparators). Conversely, the Medical Outcomes Study and BDI-II were, respectively, significantly different in comparison to the placebo and duloxetine, and to the placebo alone. Safety analysis showed a higher rate of drowsiness for the combination vs. the placebo. However, the authors highlighted that duloxetine monotherapy was not inferior to combination therapy. In this study, the authors used the 1990 American College of Rheumatology (ACR) criteria for fibromyalgia and not those of 2010 for diagnosis. Furthermore, the authors did not distinguish the outcomes for patients who started duloxetine and then added pregabalin or vice versa. This clinical investigation enrolled a small number of patients, and a slight superiority was shown only with respect to the placebo and pregabalin but not with respect to duloxetine. The main clinical trials are summarized in Table 4.

### 6.2. Safety

Tesfaye et al. [61] documented in diabetic peripheral neuropathic pain that the 8-week combination treatment with duloxetine (60 mg/day) plus pregabalin (300 mg/day) was well tolerated and safe (*p* = 0.068) vs. monotherapy.

In a multicenter, randomized, double-blind, crossover trial in patients with DPNP with a mean daily pain numerical rating scale (NRS) of 4 or higher (scale is 0–10) enrolled in 13 UK centers trial (OPTION-DM trial) [62], according to the safety analysis, the combination therapy was a safe and effective option. The most frequent side effect in the duloxetine–pregabalin group was nausea, whereas the pregabalin–amitriptyline group experienced dizziness, and the amitriptyline–pregabalin group experienced diarrhea and dry mouth.

Saxena et al. [64] did not find significant differences in terms of safety between the combination of pregabalin–duloxetine vs. pregabalin alone, with most adverse effects (such as nausea, vomiting, and somnolence) occurring during the first two weeks, before going away.

Finally, Wang et al. [65] documented that the duloxetine plus pregabalin combination group had a lower incidence of adverse events than the amitriptyline plus pregabalin group. For instance, the combination group experienced significantly less dry mouth than the amitriptyline group (12% vs. 27%), suggesting a pharmacoeconomic benefit.

## 7. Discussion

The use of combination therapy for the management of non-nociceptive pain has shown to be a reliable option according to the reported evidence. Duloxetine supplemented with pregabalin and pregabalin supplemented with duloxetine demonstrated similar effectiveness to high-dose monotherapy or other amitriptyline combination treatments, but with fewer adverse effects [61,62].

Another important observation is the evidence that combination therapy should be started gradually, titrating the first drug and then adding the second one. Tesfaye et al. showed the direct comparison of 75 patients receiving 60 mg duloxetine plus 300 mg pregabalin and 94 patients who received pregabalin 300 mg plus duloxetine 60 mg. Since the combinations did not show a statistically significant difference in terms of efficacy, the pregabalin–duloxetine group reported a higher number of total adverse events (43.6 vs. 28.0), of severe adverse events (5.3 vs. 4.0), and of adverse events leading to discontinuation (5.3 vs. 2.7) [61,66]. In consideration of these results, starting duloxetine and then adding pregabalin is the best option.

It is not futile to remember that pregabalin was the drug with the lowest rate of discontinuation due to adverse effects in clinical trials. However, the associations pregabalin plus duloxetine and duloxetine plus pregabalin may have distinct outcomes mostly in terms of safety but also efficacy; considering that duloxetine is typically linked to more adverse effects, the supplementation with pregabalin could cause a decrease in patient compliance [62,63].

The combination could be advantageous in several cases: in patients with nociplastic pain or pain associated with anxiety–depression; when patients have clinical benefit with duloxetine but without complete remission; when patients have side effects at higher dosages of duloxetine and dose reduction alone does not allow pain symptoms relief; when subjects consume other drugs that are cytochrome (CYP) 450 substrates and want to reduce duloxetine dosage; when trying another approach to central neuropathic pain (often refractory). In fact, in a recent review, Narayan et al. [67] reported a greater reduction in the mean average pain score in duloxetine-treated participants than in placebo-treated participants. One significant finding, though, was that when non-responding patients were given 120 mg of duloxetine per day, there was no discernable improvement over a dose of 60 mg. Thus, pregabalin administration may alleviate clinical symptoms in these patients.

Finally, duloxetine requires a long time to be fully effective (about 4–6 weeks) [57]; therefore, adding pregabalin (time to effective dose: 1 day) improves clinical effects without any increase in adverse reactions [68,69].

Concerning safety, pregabalin is not metabolized by CYP450, and no pharmacokinetic interactions are expected during the combination with duloxetine. Nevertheless, pharmacodynamic interactions, especially those involving the central nervous system, (CNS) may be relevant in clinical practice [2,40,70].

Choosing the proper therapeutic option is not simple. Patients with pain have no biomarkers to profile the nature of their pain. The diagnostic–therapeutic biomarkers will allow physicians to choose the most appropriate drug, for example, observing la ack of functioning in the serotonin–norepinephrine pathway or an increased release of neurotransmitters associated with pain transmitters (e.g., substance P) [71].

If the combination is considered to be easier in young patients or in subjects with few medications, it requires attention in the elderly due to polytherapy and risk of falls. Despite the low rate of pregabalin-associated interactions, it induces dizziness and may determine fractures in patients with osteoporosis, similarly to duloxetine [72,73].

The management of nociplastic pain is more complex than a simple prescription of an oral drug. It is made up of psychological support and even physical therapy [9,21,74,75]. Scientists should observe the benefits of combination therapy vs. monotherapy alongside this intervention.

This review has some limitations related to the heterogeneity of trials (enrolling patients with different diagnoses) and the scarcity of nociplastic pain data (essentially one trial). Future research must aim to conduct new randomized clinical trials in specific settings with flexible dosing schemes that need to be individualized based on patients’ characteristics, comorbidities, and medication lists. Nociplastic pain needs more evidence not only in patients with fibromyalgia but also in patients with complex regional pain syndrome and/or chronic pelvic pain syndrome.

The presence of only one clinical trial in fibromyalgia does not allow us to draw solid conclusions [66]. In this study, the effects of combination therapy on primary outcomes were comparable to those of high-dose duloxetine, suggesting that the effects of duloxetine in fibromyalgia could be related to the activity on both mood and pain. However, in this study, the superiority of the combination therapy in terms of secondary outcomes indicates a possible benefit with respect to the monotherapy.

This last point opens up an important issue. There are no biomarkers to profile the type of pain experienced by patients. Diagnostic–therapeutic biomarkers could allow physicians to choose the most appropriate therapeutic option.

Also, the follow-up should be easier. Moreover, pharmacogenomic profiling could be used on both pharmacodynamic and pharmacokinetic targets, i.e., duloxetine metabolism (CYP1A2 and CYP2D6) and pregabalin excretion and uptake in the blood–brain barrier [76,77]. Integrating cognitive behavioral therapy or psychotherapy with pharmacologic treatment in clinical trials may add strength to combination effects.

## 8. Conclusions

This is the only narrative review, to our knowledge, focusing on the combination therapy of duloxetine and pregabalin in neuropathic and nociplastic pain. The results clearly show that the combination is a reliable option for the management of neuropathic pain, whereas few data are available for nociplastic pain.

Combination therapy has proven to be as effective as high-dose monotherapy in the management of neuropathic pain, with slight differences in safety outcomes depending on supplementation, drug labels, and titration [61,62]. This evidence has been confirmed by a systematic review, and meta-analysis of combinations (opioids with antidepressants or α2δ-ligands and α2δ-ligands with antidepressants) showed no greater efficacy and found similar safety compared with each drug alone for neuropathic pain [78]. These results indicate that even if not superior, combination therapy may be an alternative to monotherapy in selected patients when the efficacy is comparable (as shown for duloxetine–pregabalin).

In nociplastic pain, the only clinical trial to our knowledge has shown superiority of pregabalin–duloxetine combination vs. pregabalin and vs. placebo, and non-inferiority vs. high-dose duloxetine. Therefore, the combination may be useful in patients who do not tolerate high-dose duloxetine [66].

Despite not being superior to monotherapy, using the combination may be advantageous in several cases: when patients have side effects at higher dosages of duloxetine and dose reduction alone does not allow symptom relief; when subjects consume other drugs that are CYP450 substrates and want to reduce duloxetine dosage; when patients with anxiety–depression experience pain that is deeply related to psychiatric symptoms; when trying another approach to central neuropathic pain (often refractory).

The final consideration is that an adequate pharmacological prescription for chronic and in nociplastic pain must nevertheless also consider the emotional and cognitive aspects of the patient that may interfere with the pharmacological therapeutic response. The poor satisfaction of patients, detected by some studies, regarding drug treatment requires attention to aspects usually overlooked (anxiety, depression, alexithymia, chronic stress, insomnia) that must be detected and considered with parallel pharmacological or psychotherapeutic interventions

## Figures and Tables

**Table 1:** Pharmacokinetic data of pregabalin and duloxetine [32,33,34].

Pharmacokinetic Parameter	Pregabalin	Duloxetine
Bioavailability (%)	≥90	32–80
Tmax (hours)	1	6
Volume of distribution (L/Kg)	0.56	23.4
Plasma protein binding (%)	None	96
Metabolism	Neglectable	CYP1A2 and CYP2D6
Excretion	Renal	Renal
Half-life (hours)	5.5–6.7	8–17

CYP, cytochrome P450; Tmax, time to peak drug concentration.

**Table 2:** Advantages and disadvantages of pregabalin and duloxetine [2,32,34,47,48].

	Pregabalin	Duloxetine
**Mechanism of action**	Action on calcium channels (α2δ subunit). Reduced release of several neurotransmitters, and reduced pain signal.	Action on serotonin and norepinephrine reuptake, increasing the modulation of pain.
**Interactions**	No interaction with cytochrome enzyme.	Interaction with cytochrome enzyme both as victim and perpetrator.
**Side effects**	Central nervous system adverse effects, like dizziness, confusion, amnesia, and drowsiness, are the most common. Label reports other clinical effects, including skin reactions, nasopharyngitis, erectile dysfunction, psychiatric and gastrointestinal side effects.	Similar to pregabalin on central nervous system. Higher rate of gastrointestinal and sexual side effects, blood hypertension. Serotonin syndrome, hepatotoxicity.
**Excretion**	Dose adjustment in patients with kidney impairment: eGFR 30–60 mL/min: 75–300 mg daily eGFR 15–30 mL/min: 25–150 mg daily eGFR < 15 mL/min: 25 mg daily	Hepatotoxic, contraindicated in patients with eGFR < 30 mL/min.
**Mood**	Indicated in patients with generalized anxiety disorder.	Anxiety–depression

CNS, central nervous system; CYP, cytochrome p450; eGFR, estimated glomerular filtration rate.

**Table 3:** Effect of pregabalin or duloxetine on non-nociceptive pain improvement with respect to control groups. NNT: number need to treat; VAS, Visual Analogue Scale.

Authors	Pain	Dosage (mg)	Score
Derry et al. [52]	Post-herpetic neuralgia	Pregabalin 300	NNT: 3.5
	Diabetic neuropathic pain	Pregabalin 600	NNT: 9.6
	Central neuropathic pain	Pregabalin 600	NNT: 5.9
Dworkin et al. [53]	Post-herpetic neuralgia	Pregabalin 300–600	Pain and sleep improvement
Migliorini et al. [54]	Fibromyalgia	Pregabalin 450	Fibromyalgia Impact Questionnaire − 1.83
Lunn et al. [55]	Diabetic neuropathic pain	Duloxetine 60–120	NNT: 5
Zhao et al. [56]	Post-herpetic neuralgia	Duloxetine 60	Pain relief (VAS score from 8 to 2); improvement in quality of life.
Birkinshaw et al. [57]	Fibromyalgia	Duloxetine 60–120	NNT: 8
Birkinshaw et al. [57]	Fibromyalgia	Duloxetine 60	Fibromyalgia Impact Questionnaire − 1.83
Finnerup et al. [39]	Neuropathic pain	Pregabalin	NNT: 6.3
	Neuropathic pain	Duloxetine	NNT: 7.7

**Table 4:** Clinical trials of pregabalin/duloxetine association. NRS, numerical rating scale; PPAR, peroxisome proliferator-activated receptor.

Authors	Pain	Dosage (mg)	Main Results
Tesfaye et al. [61]	Neuropathic pain- Diabetic neuropathy (804 patients)	Duloxetine 60 + pregabalin 300	Non-inferiority to monotherapy.
Tesfaye et al. [62]	Neuropathic pain- Diabetic neuropathy (130 patients)	Duloxetine 30 mg + pregabalin 300 150 mg twice daily	Non-inferiority to monotherapy. Increase in nausea.
Krishnaprasad et al. [17]	Neuropathic pain- Various diagnoses (328 patients)	Pregabalin 75 mg + duloxetine 30 mg	Non-inferiority to monotherapy.
Saxena et al. [64]	Neuropathic pain- Diabetic neuropathy (34 patients)	Pregabalin 75 mg + duloxetine 30 mg	Superiority to pregabalin monotherapy. Increased expression of PPAR γ (*p* < 0.001)
Wang et al. [65]	Neuropathic pain- Post-herpetic neuralgia (220 patients)	Duloxetine 30–90 mg + pregabalin 150 mg twice daily	Effective in reducing NRS. Similar efficacy when compared to amitriptyline 75 mg + pregabalin 150 mg twice daily. No comparison to monotherapy. Lower number of side effects in comparison to the other arm.
Gilron et al. [66]	Nociplastic pain- Fibromyalgia (41 patients)	Flexible dosing, ceiling: pregabalin 450 mg, duloxetine 120 mg	Superiority to placebo and pregabalin, non-inferiority to duloxetine. Higher drowsiness rate.

## Data Availability

No new data were created or analyzed in this study. Data sharing is not applicable to this article.
